# Wolves, dogs and humans in regular contact can mutually impact each other’s skin microbiota

**DOI:** 10.1038/s41598-021-96160-7

**Published:** 2021-08-24

**Authors:** Stefanie Urimare Wetzels, Cameron R. Strachan, Beate Conrady, Martin Wagner, Iwan Anton Burgener, Zsófia Virányi, Evelyne Selberherr

**Affiliations:** 1 Institute for Food Safety, Food Technology and Veterinary Public Health, Department for Farm Animal and Public Health in Veterinary Medicine, University of Veterinary Medicine Vienna, Vienna, Austria; 2 FFoQSI - Austrian Competence Centre for Feed and Food Quality, Safety & Innovation, Tulln, Austria; 3 Department of Veterinary and Animal Sciences, Faculty of Health and Medical Sciences, University of Copenhagen, 1870 Frederiksberg C, Denmark; 4 Complexity Science Hub Vienna, Josefstädter Straße 39, 1080 Vienna, Austria; 5 Small Animal Internal Medicine, Department for Small Animals and Horses, University of Veterinary Medicine Vienna, Vienna, Austria; 6 Comparative Cognition, Messerli Research Institute, University of Veterinary Medicine Vienna, Medical University of Vienna, University of Vienna, and Wolf Science Center, Domestication Lab, Konrad Lorenz Institute of Ethology, University of Veterinary Medicine Vienna, Vienna, Austria

**Keywords:** Ecology, Microbiology

## Abstract

In contrast to humans and dogs, the skin microbiota of wolves is yet to be described. Here, we investigated the skin microbiota of dogs and wolves kept in outdoor packs at the Wolf Science Center (WSC) via 16S rRNA gene amplicon sequencing. Skin swab samples were also collected from human care takers and their pet dogs. When comparing the three canine groups, representing different degrees of human contact to the care takers and each other, the pet dogs showed the highest level of diversity. Additionally, while human skin was dominated by a few abundant phylotypes, the skin microbiota of the care takers who had particularly close contact with the WSC animals was more similar to the microbiota of dogs and wolves compared to the humans who had less contact with these animals. Our results suggest that domestication may have an impact on the diversity of the skin microbiota, and that the canine skin microbiota can be shared with humans, depending on the level of interaction.

## Introduction

As domestic dogs (*Canis familiaris*) are among the most popular companion animals in Western societies, their skin microbiome, to which humans are exposed to, is of general interest^1–3^. Since domestication from wild grey wolves began more than 30,000 years ago^4^, domesticated dogs have undergone dramatic phenotypic and genotypic changes^5–8^ that are linked to having switched to living close to humans and feeding on human waste. Even though this new ecological niche of dogs has likely affected their microbiome as well, direct comparisons of the microbiota of domestic dogs and wolves (*Canis lupus*) remain at present scarce^9,10^. A few more independent investigations into taxonomic, metabolic and nutritional aspects of the canine gut microbiota and the composition of the wolf fecal microbiome have recently been published^9–13^, while that of the domesticated dogs has been studied for over a decade^14–17^ and remains of interest^18,19^. However, no information on the skin microbiota of wolves has been published and the skin microbiota of dogs has also been investigated only to a limited extent^1,3,20–23^. Importantly, the skin microbiota of dogs is of special interest, as, given that dogs and humans are frequently in physical contact and often share the same living environment, it may have a large impact on the microbiota of cohabiting humans^2^.

The skin is the interface between body and environment, is involved in regulating body temperature, and enables the sensation of touch and temperature. The skin microbiota is considered to play an important role in the prevention of disease via cross-talk with host cells, influencing cellular function and immunity^24^. It has been shown to differ greatly among body sites and individuals^1–3,22^. This has also been largely confirmed in human individuals^25–27^. Additionally, there are several additional factors known to affect the composition of the skin microbiota of humans, such as age^28,29^, birth delivery mode^30^, sex^31,32^, hygiene, geography^33–35^ and urbanization^32,36^. This is also true for the canine skin microbiota composition^20,22^. Moreover, cohabitation^2,38^ seems to be among the most important factors shaping the skin microbiota of both humans^39^ and dogs^21,22,35^, even if these changes are mostly driven by low abundant phylotypes^37^. Several studies have demonstrated that cohabitation with dogs leads to an exchange of gut as well as skin microbes between dogs and humans^2,38,40^. Sharing skin microbiota between dogs and humans has also been reported at the level of individual correlations specific to dyads living in the same household, and this process even affects the microbial exchange between cohabiting humans^2,38^. Interestingly, one study that included not only dogs but also cats as pets found weaker effects in this regard^41^, raising the possibility that dogs may have an especially strong impact on the microbiota of cohabiting humans. Given that pet owners tend to establish especially close relationships and engage in the most diverse activities with their dogs, this closeness as well as the long evolutionary history of dog–human cohabitation may contribute to the successful establishment of exchanged microbes between dogs and humans.

In the current study, we aimed to investigate the skin microbiota of wolves, dogs and humans that all had a varying amount of contact with each other and partly inhabited the same environment. To do so, we sampled dogs and wolves that had been raised by human care takers and were kept in a game park setting at the Wolf Science Center (WSC) for the purpose of behavioural and cognitive research. As such, both groups of animals had a similar but limited amount of contact to humans as compared to pets. Furthermore, the care takers of these animals and the pet dogs belonging to them were also sampled, in order to investigate whether various levels of contact with humans corresponds to different microbial compositions inhabiting the skin of composition in dogs. Indeed, the human participants and the pet dogs had varying amounts of contact to the wolves and dogs kept at the game park (WSC dogs and wolves), ranging from frequent direct contact (animal trainers) to medium or low contact (researchers studying animal behaviour). By comparing these groups of humans and canines we were able to show that the pet dogs had the highest level of diversity in their skin microbiomes. Additionally, the trainers that had particularly close contact with the animals had a more similar skin microbiome to the wolves and dogs, which was observed when comparing the distribution of various taxonomic levels. Lastly, the human skin was dominated by a few highly abundant phylotypes. Overall, our results suggest that exposure to various environments can have a large impact on the diversity of the skin microbiome and that the canine skin microbiome can be shared with humans to a degree that depends on the level of interaction.

## Results and discussion

### Humans have the least and pet dogs have the most diverse skin microbiota

**Figure 1:**
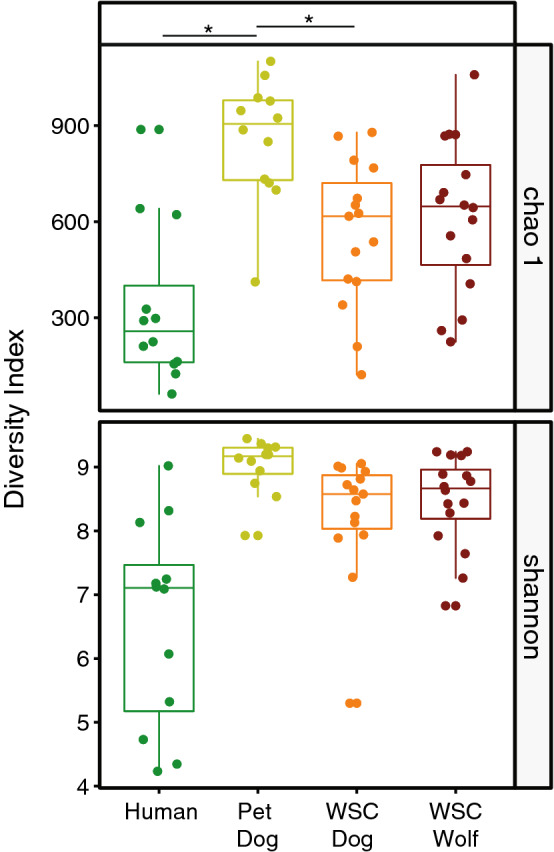
Species richness of microbiota of humans, pet dogs, WSC dogs, and WSC wolves are displayed as Chao1 index and alpha diversity as Shannon index. An asterisk indicates a *p* value below 0.05 when conducted pairwise Kruskal–Wallis test for both indices. All four groups differed significantly among each other in the pairwise comparison for the Chao1 and Shannon indices (all *p* values ≤ 0.05), except for the WSC dog group versus the WSC wolf group (both *p* values ≥ 0.05).

Out of all four groups, species richness and diversity were lowest in human skin microbiota, whereas the pet dog group had the highest species richness and diversity. All four groups differed significantly to each other (Kruskal–Wallis; Chao1, chi-squared = 20.828, df = 3, *p* < 0.001; Shannon, chi-squared = 23.332, df = 3, *p* < 0.001). In the following pairwise comparisons all groups differed significantly from each other (*p* < 0.005), except for the WSC dog group vs. the WSC wolf group, that showed a very similar species richness and diversity (Dunn's-test for multiple comparisons; Chao1, *p* = 0.574; Shannon, *p* = 0.579; Fig. 1.). The low species richness and diversity in the human skin microbiota has also been shown in previous studies^42,43^ and might be driven by physiological differences of the skin between humans and canines, such as pH and hair covering, as well as by skin hygiene practices and differences in regular environmental contact^44,45^. For humans, protection from invasion by microorganisms is controlled by microbial desiccation, competition with resident microbiota, and an acidic pH. The average reported cutaneous pH of humans is 4.8, while the average cutaneous pH of dogs is 7.4, suggesting that the higher pH might support a more complex skin microbiota composition, as compared to humans^45^. Moreover, Clemente et al.^33^ showed that the skin microbiome of uncontacted humans living outdoors is significantly more diverse than that of westernized people, supporting the assumption that modern habits, including personal and environmental hygiene, lead to a decrease in skin microbiota species richness and diversity^33,46^. With respect to the high diversity of the pet dogs’ microbiota, we suggest that the pet dog skin was regularly exposed to a diverse set of environmental microbiomes, both indoors and outdoors. The WSC animals (wolves and dogs), in contrast, are restricted to the game park environment.

### Humans and pet dogs are less similar compared to WSC dogs and wolves, which are more similar to each other in their skin microbiota composition

**Figure 2:**
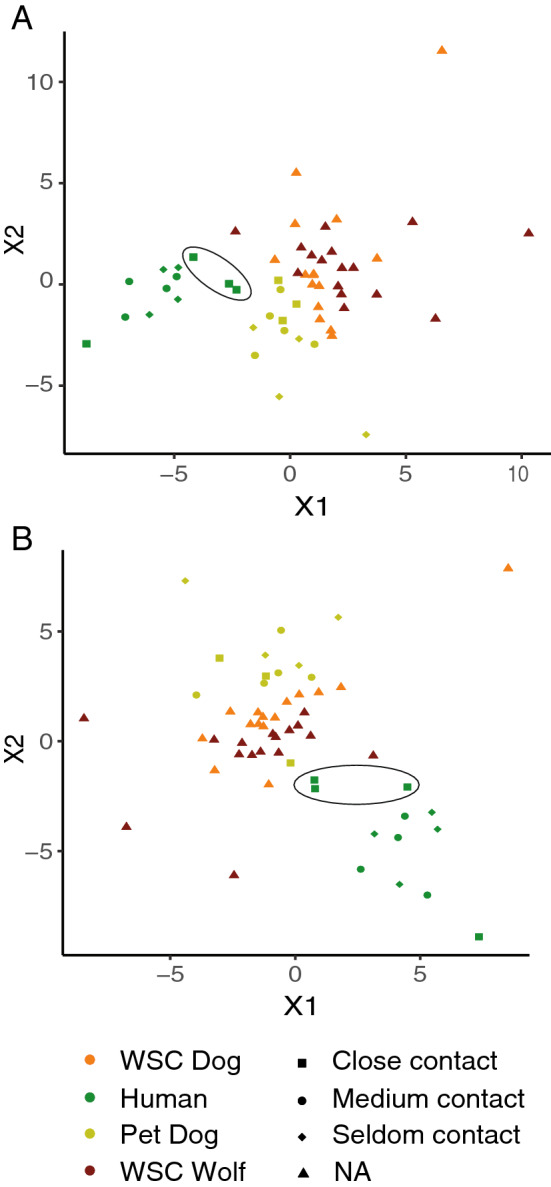
t-distributed stochastic neighbor embedding (tSNE) plots. Humans that have very close and frequent physical interactions with the WSC animals (dog and wolf group) are circled and more similar to the WSC animals. (**A**) Jensen–Shannon divergence. (**B**) Bray–Curtis dissimilarity.

The analysis of independent variable influence, i.e. sex (male vs. female), age class (sub adult vs. adult), last antibiotic treatment (early vs. late) and human contact did not reveal significant effects on the canine skin microbiota composition (multivariate PERMANOVA in Adonis; diet, *p* = 0. 387; age, *p* = 0.136; sex, *p* = 0.114; last antibiotic treatment, *p* = 0.728, human contact, *p* = 0.539), but the groups differed significantly (multivariate PERMANOVA in Adonis; group, R^2^ = 0.074, F model = 1.622, *p* = 0.0228). Also in beta diversity significant differences were detected between groups (PERMANOVA; Bray–Curtis dissimilarity; pseudo R^2^ = 0.180; F model = 3.741; *p* = 0.001). In the pairwise comparison, all groups differed significantly from each other (R^2^ = 0.076–0.209, F model = 1.468–6.598, *p* = 0.006). In the visual inspection of tSNE plots which were based on Jensen-Shannon divergence and Bray–Curtis dissimilarity (Fig. 2.), humans and pet dogs appear less similar to each other and the other groups, while WSC dogs and wolves appear more similar to each other in their skin microbiota composition. Not surprisingly, among the four groups humans appeared most distinct to all canines that grouped more closely together.

Physiological differences between canine species affecting their microbiota may be relatively small. DeCandia et al. (2019)^47^ found out that coyotes, red and grey foxes living in the wild in North-America responded with a similar microbial community shift to a *Sarcoptes scabiei* mite infection. However, the environment and living conditions of individuals do lead to a difference in microbial composition within species^48^ suggesting that environmental effects on microbiota are significant. These observations are also consistent with the pairwise alpha diversity comparisons above, where the only non-significant difference between groups was the comparison of WSC dogs to wolves.

While physiological differences of the skin are likely to contribute to the differences observed between humans and canines, in this study mutual contact and living in the same environment have likely reduced difference between the canine groups^44,45^. This is probably an important factor that has made the WSC dog and wolf samples most similar to each other (Fig. 2.). This is consistent with the species richness and alpha diversity, and suggests that environmental exposure has a similar or stronger impact on shaping the skin microbiota than the evolutionary segregation of wolves and dogs due to domestication (see also^21,22,35^).

It has further been suggested that diet shapes the skin microbiome^49^. Here, diet does not explain the difference between the pet dog group and the WSC animals (PERMANOVA; diet, R^2^ = 0.046, F model = 1.007, *p* = 0.386). Both dog groups, WSC dogs and pet dogs were fed a similar diet. However, the WSC dog and wolf groups microbial community structure was similar to each other despite being fed different diets (Supplementary Dataset 1).

The hologenome theory of evolution supports the idea that specific groups or species evolved together with their microbiomes, and that this symbiosis greatly affects their health status^50,51^. A loss of microbiome diversity can be caused by several factors and may impact health. For example, a decrease in species richness and diversity can be caused by specific living conditions over several generations, as seen in both humans and canines living in or close to urban environments, as compared to populations living under natural conditions^33^. Outside of these long-term shifts in the skin microbiomes of certain groups, similar changes in diversity that do not necessarily impact health, can also be caused within shorter time spans, as represented in our canine groups. The pet dogs within the current study were similar to each other in terms of beta diversity, but distinct from the WSC animals (Fig. 2). Of the pet dogs, four out of twelve were originally born at the WSC and later on adopted by the human caretakers, while remaining on the same diet; their origin however apparently did not leave remaining track in their microbiome as they were not more similar to the WSC dogs than the other pet dogs. Lastly, three of the four humans, that were a priory classified as having close physical interactions with the WSC dogs and wolves were more similar to the WSC animals than the other human participants with less animal contact (PERMANOVA; R^2^ = 0.167, F = 1.967, *p* = 0.077; Fig. 2). This was also found when conducting a PCoA (Supplementary Fig. 1). Based on this finding, a new group named “human close” was used to label downstream analyses and included the three human samples that clustered closer to the animals.

### The skin microbiota of humans with close contact to WSC animals is more similar to the microbiota of the WSC animals

**Figure 3:**
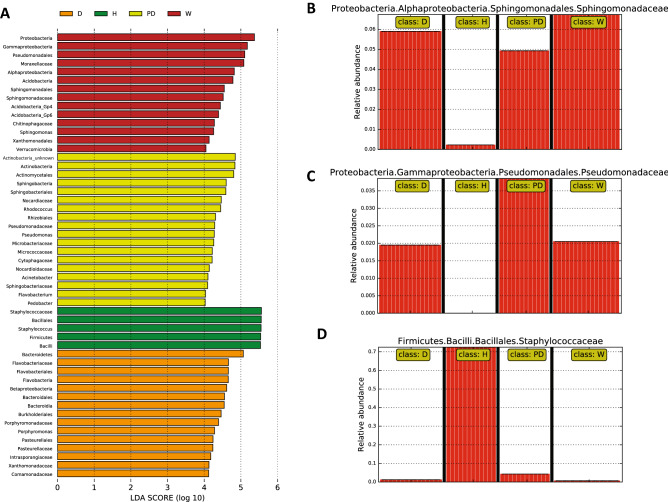
(**A**) LEfSe (Linear discriminant analysis Effect Size) calculation which determines features most likely to explain differences between groups, (**B**–**D**) LEfSe plot on differential features. H = human, PD = pet dog, D = WSC dog, W = WSC wolf.

**Figure 4:**
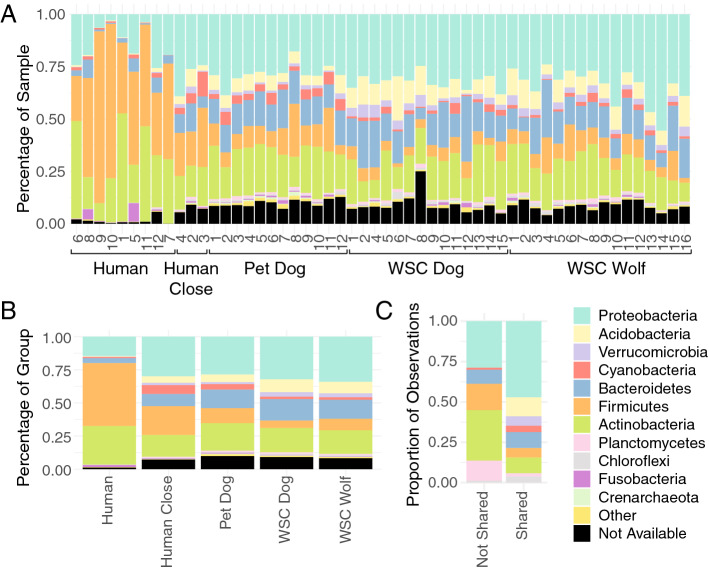
Phylum level taxonomic distributions. (**A**) Each individual sample comparison ordered by group. Humans are additionally ordered by level of animal contact, as classified based on the tSNE plots (Fig. 2). (**B**) Average distribution across groups, including humans with close contact. (**C**) Proportion of ASVs that are exclusively observed more than 200 times in only non-close contact Humans (not shared) or in both close contact Humans and Pet Dogs (shared).

LEfSe analysis revealed an enrichment of *Acidobacteria, Verrucomicrobia* and *Proteobacteria* (in particular *Alphaproteobacteria* and *Gammaproteobacteria*) in wolves, and *Bacteroidetes* and *Proteobacteria* (in particular *Betaproteobacteria*) in WSC dogs, whereas *Firmicutes* were enriched in human samples (Fig. 3A). Interestingly, not only the three canine groups, but also the humans with close contact to the WSC animals, had a microbiome that showed higher proportions of *Proteobacteria*, *Acidobacteria*, *Verrucomicrobia*, and *Bacteroidetes* (Fig. 4). The proportions of *Firmicutes* and *Actinobacteria* were lower in these groups compared to the humans without close contact to the WSC animals. This suggests that contact with the animals increased the ratio of gram negative to gram positive microorganisms on the skin, and the phylum level diversity. The *Proteobacteria* appeared to be particularly shared between humans with close animal contact and pet dogs (Fig. 4). The patterns observed at phylum level could also be seen on lower taxonomic levels in the LEfSe analysis, with a significant enrichment of f.e. *Sphingomonadaceae* in the wolf group (Fig. 3B), and f.e. *Pseudomonadaceae* in the WSC dog group (Fig. 3C). Consistent with this observation, humans with close contact to the WSC animals had microbial community shifts mainly caused by an increase in *Pseudomonadaceae*, *Sphingomonadaceae* and *Flavobacteriaceae* abundance and a decrease of *Staphylococcacceae* and *Corynebacteriaceae* abundance (Supplementary Fig. 2).

Lehtimaki et al.^39^ found that intensive contact to forest and arable land (which was the case for our canine groups compared to the human individuals) correlated with a higher diversity of soil-based *Proteobacteria* and other soil-related taxa on skin, while in urban areas, skin-based *Actinobacteria* were more abundant. This was also the case in our human samples, with the exception of those from humans with very close contact to WSC animals. This supports the importance of human-animal interactions in providing exposure to environmental microbes and affecting the skin microbiome composition^2,23^. These findings also indicate that not only the pet dog skin microbiota seems to be greatly affected by their surroundings, but that the human skin microbiota can also show strong shifts when being exposed to canine animals and their environment. Coelho et al.^53^ recently indicated that the similarities in the gut microbiome of dogs and humans might not be solely explained by direct transmission, but rather a function of similar physiology and lifestyle^53^. Lehtimaki et al.^54^ also examined that a shared living environment as well as lifestyle patterns correlate with microbial similarities in dog-owner pairs and that they influence the structure of skin microbiota in both species. In our study, direct transmission might have also contributed to the similarities observed, as the skin was not cleaned before sampling thereby washing off allochthonous microorganisms, as applied in skin microbiota sampling of amphibians^43,55^. However, different to former studies on pets^2,38^, we found no evidence that owners and pet dogs living in the same household would have more similar skin microbiota to each other than to other pet dogs or other owners, respectively.

A rural environment or countryside lifestyle are known to boost protection against allergic diseases^39^. This protection has suggested to be mediated by acquiring a more diverse microbiome or by exposure to environmental microbes that support immune tolerance^39^. In this study, the three humans with close contact to the WSC animals had a microbiota structure with altered composition (PERMANOVA; R^2^ = 0.167, F = 1.967, *p* = 0.077) but without higher species richness and/or diversity. Whether these changes might benefit their hosts in terms of immune responses or allergies, as suggested previously^39,46,54^, needs further investigation.

### The human skin microbiota

A total of 15,181 ASVs were detected in all skin samples from the four groups. In the human group, 4,676 ASVs were found, of which 581 (12%) were shared with the pet dogs. *Firmicutes*, in particular *Staphylococcaceae* were identified as significantly enriched in humans in the LefSe analysis (Fig. 3A,D). The six most abundant ASVs in the human group were classified as *Staphylococcus* with relative abundances from 7.22 to 2.39%. The dominance of *Staphylococcaceae* is in line with previous literature^56^. *Staphylococcus* species are known to especially inhabit the human skin as commensals and opportunistic pathogens^57^. Other highly abundant phylotypes detected in the current dataset have also been described as normal inhabitants of the human skin, such as *Corynebacteria*, that show a higher sensitivity to environmental factors compared to staphylococci^58^. *Cutibacterium* has also been found on human skin samples. *Cutibacterium acne* is seen as an important species within this genus, which is linked to acne and balanced by specific *Staphylococcus* strains^59^. Several ASVs among the 50 most abundant, which were classified as *Staphylococcus, Corynebacterium* and *Cutibacterium* were significantly higher abundant in human samples in the pairwise comparison with canine skin samples (Table 1).

**Table 1:** The statistically significant differences in relative abundance among the 50 most abundant ASVs are shown for the human, pet dog, WSC dog und wolf group.

Taxonomy	ASV number	Relative abundance ± standard deviation [%]			
		Human	Pet Dog	WSC Dog	WSC Wolf
*Staphylococcus*	1	7,22 ± 6,700^a^	0,27 ± 0,173^b^	0,09 ± 0,345^c^	0,00 ± 0,000^c^
*Staphylococcus*	2	5,19 ± 4,930^a^	0,18 ± 0,130^b^	0,01 ± 0,055^c^	0,03 ± 0,132^c^
*Staphylococcus*	3	4,58 ± 4,227^a^	0,12 ± 0,154^b^	0,00 ± 0,000^b^	0,00 ± 0,000^b^
*Staphylococcus*	4	4,12 ± 3,960^a^	0,08 ± 0,090^b^	0,00 ± 0,000^c^	0,00 ± 0,000^c^
*Rhodococcus*	5	0,50 ± 0,759^b^	0,48 ± 0,522^ab^	1,26 ± 0,892^a^	0,83 ± 0,849^ab^
*Pseudomonas*	6	0,75 ± 1,455	0,52 ± 1,225	0,31 ± 0,469	1,10 ± 2,541
*Staphylococcus*	7	2,46 ± 2,434^a^	0,03 ± 0,051^b^	0,00 ± 0,000^b^	0,00 ± 0,000^b^
*Staphylococcus*	8	2,39 ± 2,409^a^	0,01 ± 0,034^b^	0,00 ± 0,000^b^	0,00 ± 0,000^b^
*Cutibacterium*	9	2,21 ± 2,576^a^	0,00 ± 0,000^b^	0,02 ± 0,067^b^	0,00 ± 0,000^b^
*Sphingomonas*	10	0,14 ± 0,208^b^	0,46 ± 0,401^ab^	0,63 ± 0,370^a^	0,60 ± 0,530^a^
*Streptococcus*	11	1,94 ± 3,045^a^	0,14 ± 0,125^b^	0,01 ± 0,036^c^	0,01 ± 0,024^c^
*Staphylococcus*	12	1,78 ± 1,848^a^	0,01 ± 0,018^b^	0,00 ± 0,000^b^	0,00 ± 0,000^b^
Unclassified *Micrococcaceae*	13	0,06 ± 0,119^b^	0,35 ± 0,213^a^	0,60 ± 0,386^a^	0,47 ± 0,359^a^
Unclassified *Xanthomonadaceae*	14	0,11 ± 0,230^b^	0,35 ± 0,342^ab^	0,39 ± 0,433^ab^	0,59 ± 0,498^a^
*Rhodococcus*	15	0,12 ± 0,274^b^	0,21 ± 0,335^ab^	0,64 ± 0,581^a^	0,43 ± 0,603^ab^
*Rhodococcus*	16	0,14 ± 0,255	0,21 ± 0,270	0,59 ± 0,500	0,42 ± 0,549
*Micrococcus*	17	1,09 ± 1,244^a^	0,49 ± 0,492^a^	0,03 ± 0,109^b^	0,02 ± 0,064^b^
*Pseudomonas*	18	0,33 ± 0,821	0,22 ± 0,510	0,14 ± 0,263	0,67 ± 1,451
*Cutibacterium*	19	1,54 ± 1,745^a^	0,01 ± 0,013^b^	0,00 ± 0,000^b^	0,00 ± 0,000^b^
*Pseudomonas*	20	0,33 ± 0,734	0,20 ± 0,506	0,10 ± 0,193	0,63 ± 1,433
*Staphylococcus*	21	0,00 ± 0,000	0,07 ± 0,187	1,11 ± 4,252	0,01 ± 0,053
Gp4	22	0,02 ± 0,086^c^	0,06 ± 0,131^bc^	0,74 ± 0,549^a^	0,31 ± 0,318^ab^
*Janibacter*	23	0,11 ± 0,282^b^	0,06 ± 0,152^b^	0,54 ± 0,591^a^	0,40 ± 0,703^ab^
*Rhodococcus*	24	0,15 ± 0,289	0,20 ± 0,273	0,50 ± 0,439	0,31 ± 0,451
*Streptococcus*	25	1,31 ± 1,875^a^	0,03 ± 0,079^b^	0,02 ± 0,077^b^	0,00 ± 0,000^b^
*Cutibacterium*	26	1,36 ± 1,472^a^	0,00 ± 0,000^b^	0,00 ± 0,000^b^	0,00 ± 0,000^b^
Unclassified *Microbacteriaceae*	27	0,08 ± 0,209^b^	0,41 ± 0,242^a^	0,43 ± 0,313^a^	0,24 ± 0,294^ab^
*Massilia*	28	0,13 ± 0,218^a^	0,19 ± 0,223^ab^	0,54 ± 0,489^a^	0,25 ± 0,227^ab^
*Pseudomonas*	29	0,28 ± 0,690	0,15 ± 0,417	0,15 ± 0,233	0,51 ± 1,230
*Corynebacterium*	30	1,19 ± 2,431^a^	0,09 ± 0,143^b^	0,01 ± 0,020^b^	0,00 ± 0,000^b^
*Nocardioides*	31	0,07 ± 0,226	0,36 ± 0,421	0,45 ± 0,447	0,23 ± 0,290
Unclassified *Intrasporangiaceae*	32	0,19 ± 0,299	0,48 ± 0,501	0,27 ± 0,537	0,18 ± 0,221
*Cutibacterium*	33	1,22 ± 1,338^a^	0,01 ± 0,017^b^	0,00 ± 0,000^b^	0,00 ± 0,000^b^
*Spartobacteria_genera_incertae_sedis*	34	0,08 ± 0,195	0,03 ± 0,079	0,47 ± 0,587	0,34 ± 0,666
*Sphingomonas*	35	0,05 ± 0,190^b^	0,35 ± 0,555^ab^	0,42 ± 0,434^a^	0,14 ± 0,263^ab^
*Pedobacter*	36	0,00 ± 0,000^c^	0,45 ± 0,379^a^	0,43 ± 0,479^ab^	0,08 ± 0,138^bc^
Unclassified *Actinomycetales*	37	0,96 ± 2,368^a^	0,11 ± 0,124^a^	0,01 ± 0,032^b^	0,00 ± 0,000^b^
*Massilia*	38	0,13 ± 0,192	0,20 ± 0,219	0,29 ± 0,301	0,28 ± 0,360
Gp16	39	0,06 ± 0,206	0,28 ± 0,367	0,32 ± 0,408	0,22 ± 0,330
Unclassified *Pasteurellaceae*	40	0,12 ± 0,338^b^	0,34 ± 0,237^a^	0,24 ± 0,274^ab^	0,19 ± 0,313^ab^
*Acinetobacter*	41	0,30 ± 0,711	0,15 ± 0,263	0,16 ± 0,245	0,27 ± 0,258
*Polaromonas*	42	0,00 ± 0,000	0,32 ± 0,440	0,21 ± 0,400	0,30 ± 0,374
*Sphingosinicella*	43	0,02 ± 0,082^c^	0,09 ± 0,208^bc^	0,30 ± 0,358^ab^	0,37 ± 0,377^a^
*Sphingomonas*	44	0,04 ± 0,087^b^	0,24 ± 0,146^a^	0,36 ± 0,317^a^	0,17 ± 0,230^ab^
Unclassified *micrococcaceae*	45	0,16 ± 0,255	0,22 ± 0,198	0,12 ± 0,157	0,31 ± 0,210
Unclassified *intrasporangiaceae*	46	0,10 ± 0,332	0,25 ± 0,336	0,23 ± 0,363	0,21 ± 0,422
*Cryobacterium*	47	0,15 ± 0,284	0,29 ± 0,328	0,09 ± 0,180	0,27 ± 0,296
*Sphingomonas*	48	0,00 ± 0,000^c^	0,27 ± 0,361^ab^	0,08 ± 0,195^bc^	0,38 ± 0,404^a^
*Acinetobacter*	49	0,06 ± 0,146	0,10 ± 0,174	0,25 ± 0,519	0,29 ± 0,570
*Janibacter*	50	0,10 ± 0,210	0,06 ± 0,141	0,30 ± 0,504	0,25 ± 0,347

Different superscripts in same row indicate statistical significance (*p* value ≤ 0.05).

### The canine skin microbiota

This is the first study to describe the bacterial composition on the skin of wolves. Overall, the wolf skin was inhabited by similar abundant phylotypes compared to the dog skin. The most abundant ASVs in the three canine groups were classified as *Pseudomonas*, *Rhodococcus*, *Staphylococcus*, *Micrococcus* and *Sphingomonas* with relative abundances between 1.26 to 0.60% (Table 1, Supplementary Dataset 2). Several phylotypes were enriched in the wolf group in the LEfse analysis, among them *Alpha-* and *Gammaproteobacteria*, *Sphingomonadaceae* and *Pseudomonadales*. In the WSC dog group, *Bacteroidetes, Betaproteobacteria* and *Flavobacteriaceae* were enriched, while in the pet dog group *Actinobacteria, Pseudomonadaceae* and *Sphingobacteria* showed significant enrichment (Fig. 3). Only one of the 50 most abundant ASVs that differed significantly between the WSC wolf and WSC dog group (*Sphingomonas* ASV 48*,* enriched in WSC wolf skin samples compared to WSC dog skin samples), while seven ASVs differed significantly between the WSC wolf and pet dog groups (three *Staphylococcus*-ASVs, *Streptococcus*, *Pedobacter,* unclassified *Actinomycetales* and *Sphingosinicella*, Table 1). Except of *Sphingosinicella* all of these ASVs were enriched in the pet dog skin samples, compared to the WSC wolf skin samples. All highly abundant phylotypes have been found on the skin of healthy dogs before^2^. Overall, these findings indicate that the wolf skin microbiota is similar to the dog skin microbiota, but several phylotypes are more dominant in the WSC animals. This might be largely affected by living environment^21^, but whether and to what extent the similar skin microbiota is due to the wolf and dog subjects of the current study sharing the same environment is difficult to tell. A former study on red wolves has shown that living in a captive environment significantly affects the gut microbiota of the animals, and this effect is apparent even if the animals are fed with a diet reflective of their natural environment^60^. Given that our wolves also live in captivity and do share some of the facilities with the dogs, it is not surprising that the highly abundant ASVs on the WSC wolf skin were also present on the WSC dog skin. However, the fact that they were detected also on the skin of the pet dogs in this study suggests fundamental similarities in the skin microbiota of wolves and dogs, although the abundances of these phylotypes differed among groups. In this study, the shared environment of the wolves and WSC dogs seem to have impacted at least the profile of highly abundant phylotypes of the animals’ skin microbiota (Table 1, just 1 ASV was significantly different between WSC dogs and wolves) and the WSC groups had a similar alpha diversity (WSC dog group vs. the wolf group; Dunn's-test for multiple comparisons; Chao1, *p* = 0.574; Shannon, *p* = 0.579). The shared environment might have had more impact on the alpha diversity than their differential diet and their divergent evolution, as also indicated by the lower similarity between the wolf and pet dog group (Dunn’s test; Chao1, *p* = 0.025; Shannon, *p* = 0.041) compared to the wolf and WSC dog group (Dunn’s test; Chao1, *p* = 0.574; Shannon, *p* = 0.579). The number of shared ASVs within the groups is shown in Supplementary Fig. 3. A huge amount of low abundant organisms was uniquely found in each canine group. The number of unique ASV was more than double in canine groups compared to the humans. The importance of a high skin microbiota diversity in canines, the function of unique low abundant phylotypes, as well as the importance of specific microbial enrichments in groups has not been investigated until now and has to be examined in future research.

## Conclusion

Overall, the current results suggest that exposure to various environments and cohabitation affect the skin microbiota more than ecological and physiological changes that took place during the course of dog domestication and can lead to a significant shift of the skin microbiome both in dogs and in humans. Close contact with dogs and wolves shape the human skin microbiome by causing large shifts in the microbiota composition at the phylum and family level, leading to an increase in the ratio of gram negative to positive bacteria.

## Material and methods

### Ethics statement

The study was discussed and approved by the institutional ethics and animal welfare committee of the University of Veterinary Medicine, Vienna in accordance with good scientific practice guidelines and Austrian national legislation TVG 2012 (ETK-32/02/2019). The study was discussed and approved by the ethics committee of the Medical University, Vienna in accordance with good scientific practice guidelines (EK Nr.: 1058/2020). Informed consent was obtained from all human participants.

### Sample collection

Skin swab samples were collected from 16 wolves and 15 dogs kept in large enclosures at the Wolf Science Center (WSC) in Ernstbrunn, Austria. In addition, skin swabs were also taken from 12 humans working at the WSC and 12 pet dogs living in the households of ten of these 12 human individuals. The WSC animals (wolf and dog groups) were hand-raised from their age of 1 week to 5 months of age when they were introduced in packs of adult animals. This period included 20–24 h of close contact to humans daily, including bottle- and hand-feeding, and sleeping together (for more see Virányi and Range (2011)^64^). Later on, the WSC animals participated in daily sessions of training, along with cognitive and behavioral testing, that included direct physical contact with their trainers. The pet dogs lived in the households of ten of the human participants of the study, had regular access to the WSC environment and were, to varying degrees, in direct contact with the WSC dogs and wolves. A complete and detailed list of all individuals including information about diet, age, sex, origin, health status of the sampled individuals, dog-owner relationship and degree of contact with WSC animals is described in Supplementary Dataset 1, where sample IDs were assigned randomly. The skin swab samples from all individuals (humans, pet dogs, WSC dogs and WSC wolves) were taken from the chest region by rotating the swab (Raucotupf Cotton tipped applicators “S”, Lohmann & Rauscher GmbH, Austria, Vienna) ten seconds on the skin. Blinding during sample collection was not possible. Sampling of all groups happened within the same 3 month period in autumn. Samples were frozen at − 20 °C within 15 min after sampling and stored at − 20 °C until further processing.

### DNA extraction and illumina 16S rRNA gene amplicon sequencing

The samples were thawed at room temperature immediately before DNA extraction. For DNA isolation the DNeasy PowerSoil Kit (Qiagen, Hilden, Germany) was used according to the manufacturers ‘ protocol, with the exception of mechanical lysis, which was conducted for 20 min instead of 10 min. DNA was eluted in 50 µl DEPC-treated water preheated to 70 °C. DNA concentration was determined using the Qubit 2.0 Fluorimeter (Qubit dsDNA BR Assay Kit, Thermo Fisher Scientific, Vienna, Austria). The samples were all processed at the same time in two batches and a negative extraction control was isolated and processed together with each batch of skin swab samples. For sequencing of 56 samples (skin swab samples plus one negative extraction control), the hypervariable region V4 of bacterial and archaeal 16S rRNA genes was targeted using the primer set 515F-GTGYCAGCMGCCGCGGTAA-modified^65^ and 806R-GGACTACNVGGGTWTCTAAT-modified^66^ to generate an approximate amplicon size of 291 bp. Libraries were constructed by ligating sequencing adapters and indices onto purified PCR products using the Nextera XT Sample Preparation Kit (Illumina) according to the recommendations of the manufacturer (including negative controls for the PCR). Equimolar amounts of each of the libraries were pooled and submitted for sequencing on an Illumina MiSeq Personal Sequencer using a 300 bp read length paired-end protocol (MiSeq Reagent Kit v3). Sequencing was performed at the Core Facility Molecular Biology of the Medical University Graz, Austria and sequencing data was provided as demultiplexed forward and reverse fastq files.

### Read processing

For all analyses, the forward reads from the dataset were processed into amplicon sequence variants (ASV) using DADA2^67^ (version 1.9.1) with a forward cutoff of 30 and a length cutoff of 290 within the QIIME2^68^ (version 2019.1) environment. All ASVs present in the negative control above 3% relative abundance were removed, leading to the removal of 10 ASVs. Before all beta-diversity and taxonomic comparisons were made, sequences that were classified at the class level as ‘Chloroplast’ were removed. A full overview on the absolute abundances per sample is shown in Supplementary Dataset 3. This data was used for all alpha and beta- diversity metrics, in addition to phylum level comparisons. Phylum level taxonomic assignment was conducted using the RDP package within R (version 1.20.0).

The python and R code used in the analysis is available at [https://github.com/cameronstrachan/WolfSkinCommunity2020].

### Statistical analysis

Only samples with a total of more than 10,000 reads after quality control were kept. Relative abundances were then calculated and used in downstream statistical analysis. Permutational multivariate analysis of variance (PERMANOVA) was used to examine the association between the microbial communities and independent variables including sex, diet, age, last antibiotic treatment and human contact. Species richness were calculated using the Chao1 index and Shannon index. The normality distribution of alpha diversity and ASVs was checked in semi-parametric factorial designs with corresponding histograms, Q–Q plots, residual versus fitted plots, the Shapiro–Wilk Test and skewness and kurtosis test for normality as well as for different transformations techniques such as log transformation. Additionally, the homogeneity of variances across samples was tested with the Levense’s test. Due to the non-normal distribution of diversity data and ASV abundances, the Kruskal–Wallis test was applied followed by a Dunn’s test for multiple comparisons. Further, means, standard deviations, and standard errors were calculated. The results were considered statistically significant at *p* value < 0.05 and the *p* value was adjusted for multiple comparison with the p-adjusted method Benjamini & Hochberg. The statistical analysis was done using the R statistical computing environment (Version 4.0.5 R Foundation for Statistical Computing, Vienna, Austria) using the package normtest, and stats and vegan. For beta diversity analysis, T-distributed stochastic neighbor embedding (tSNE) plots, based on Jensen-Shannon divergence and Bray–Curtis dissimilarity, were calculated with the package tsne. To find biomarkers that explain the differences between the groups, LEfSe^69^ was applied by using default values, except of the threshold on the logarithmic LDA score for discriminative features, which was set to 4.

### Accession number

Illumina MiSeq sequencing data are available in BioProject SRA database under the accession number PRJEB36827.

### ARRIVE guidelines

The study was carried out in compliance with the ARRIVE guidelines (http://www.nc3rs.org.uk/page.asp?id=1357).

## Supplementary Information

Supplementary Figure 1.

Supplementary Figure 2.

Supplementary Figure 3.

Supplementary Dataset 1.

Supplementary Dataset 2.

Supplementary Dataset 3.
