# Supplementary figures and images for: Wolves, dogs and humans in regular contact can mutually impact each other’s skin microbiota
Source: Sci Rep. 2021 Aug 24;11:17106. doi: 10.1038/s41598-021-96160-7 (PMC8385068; doi:10.1038/s41598-021-96160-7)

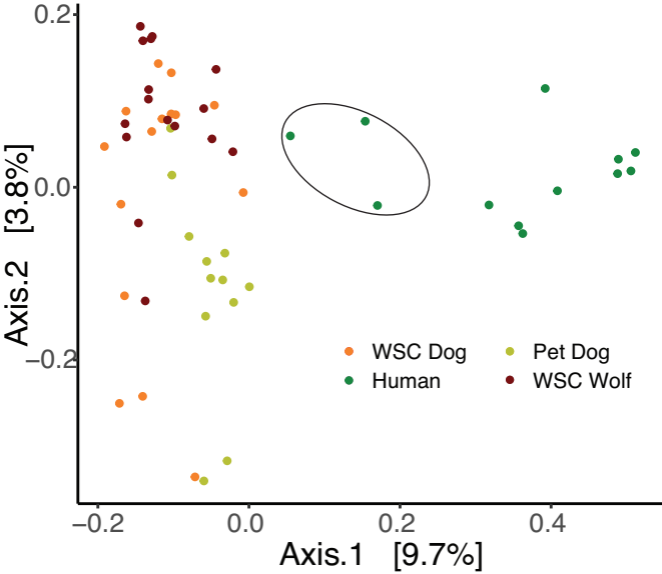

Supplement: Supplementary file 1 — Supplementary Figure 1. [file 41598_2021_96160_MOESM1_ESM.pdf]

**A**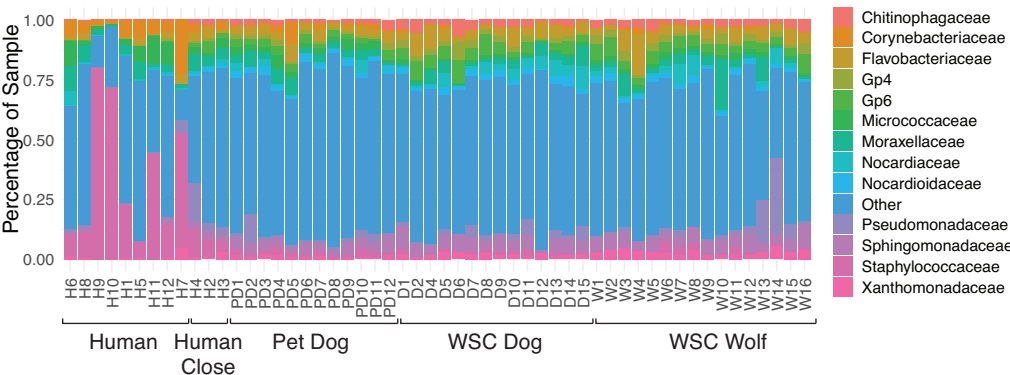**B**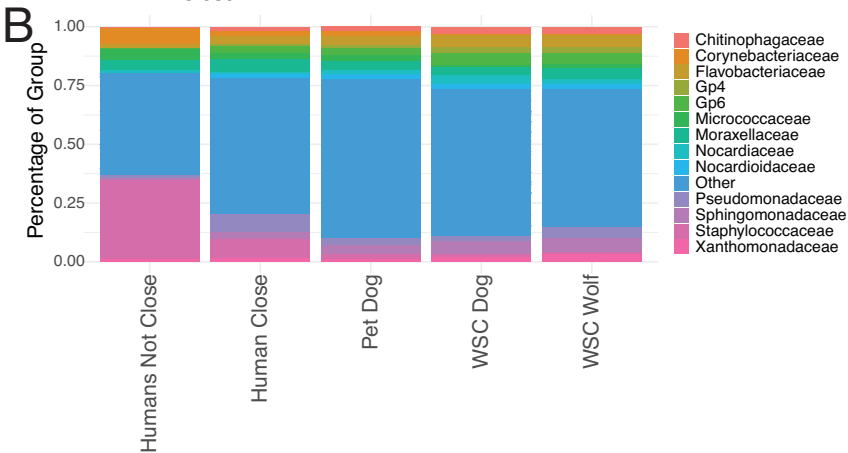

Supplement: Supplementary file 2 — Supplementary Figure 2. [file 41598_2021_96160_MOESM2_ESM.pdf]

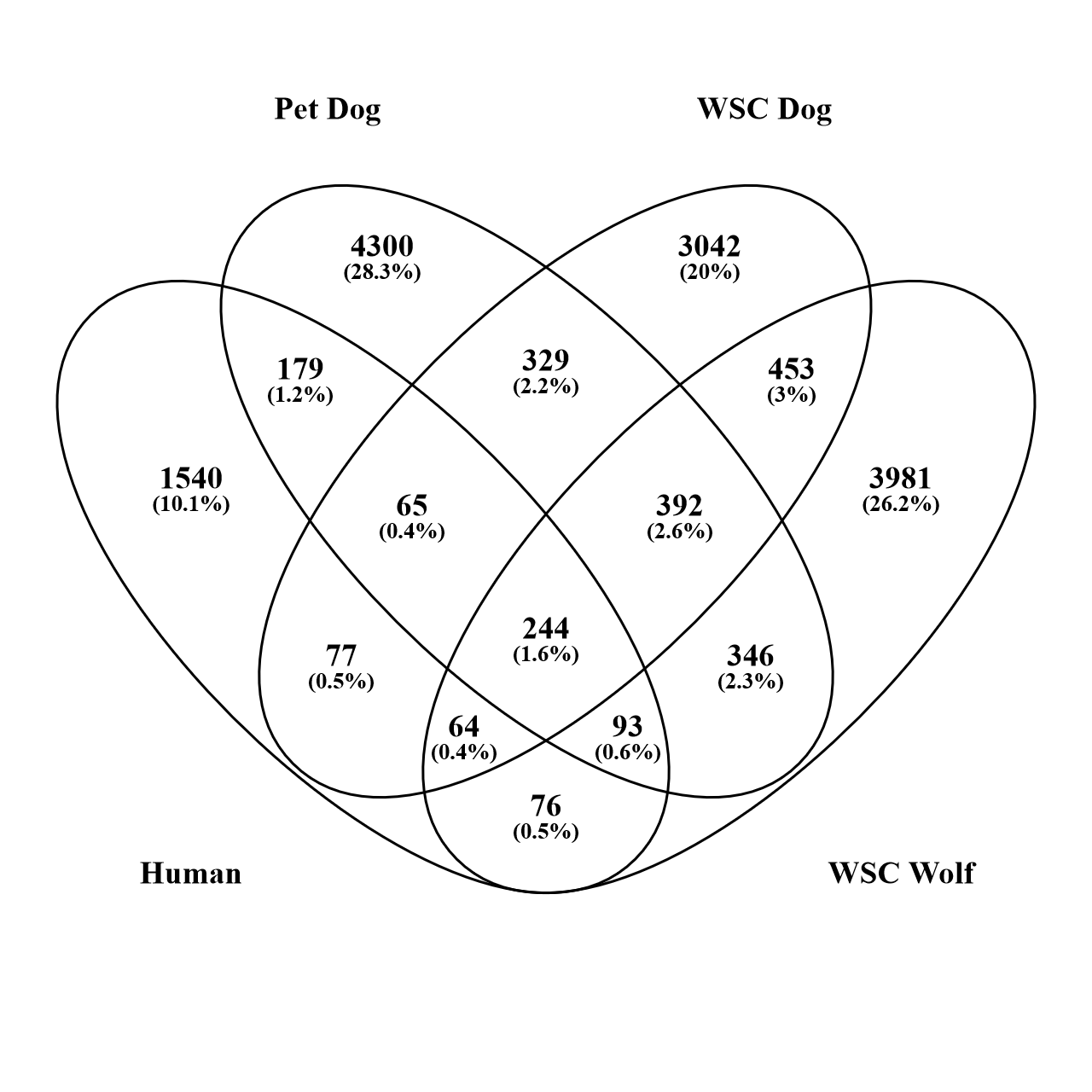

Supplement: Supplementary file 3 — Supplementary Figure 3. [file 41598_2021_96160_MOESM3_ESM.png]
